# Supplementary material for: Impact of concurrent aerobic and resistance training on body composition, lipid metabolism and physical function in patients with type 2 diabetes and overweight/obesity: a systematic review and meta-analysis
Source: PeerJ. 2025 Jun 11;13:e19537. doi: 10.7717/peerj.19537 (PMC12166852; doi:10.7717/peerj.19537)
Supplement: Supplemental Information 2 — The databases (e.g., PubMed, Scopus, Google Scholar, Cochrane Library) and the specific search algorithms applied to identify studies related to exercise, training, and type 2 diabetes. [file peerj-13-19537-s002.docx]

**Table S1.** Search strategy

| **#** | **Database** | **Algorithm** |
| --- | --- | --- |
| 1 | PubMed | ("Exercise"[Title/Abstract] OR "training"[Title/Abstract]) AND ("diabet*"[Title/Abstract]) |
| 2 | Scopus | Title- ABS (exercise OR training) AND Title- ABS (diabetes) |
| 3 | Google Scholar | Allintitle (Exercise OR Training) ( type 2 diabetes) |
| 4 | Cochrane Library | (Exercise OR Training) (type 2 diabetes) |
| 5 | Web of Science | (ALL Exercise OR Training) AND (type 2 diabetes) |
| 6 | Science Direct | (Exercise OR Training) (type 2 diabetes) |
| 7 | SPORT Discus | (Exercise OR Training) (type 2 diabetes) |
