# Supplementary material for: Impact of concurrent aerobic and resistance training on body composition, lipid metabolism and physical function in patients with type 2 diabetes and overweight/obesity: a systematic review and meta-analysis
Source: PeerJ. 2025 Jun 11;13:e19537. doi: 10.7717/peerj.19537 (PMC12166852; doi:10.7717/peerj.19537)
Supplement: Supplemental Information 3 — The quality and potential biases in each study, categorized into domains such as random sequence generation, allocation concealment, blinding of participants and personnel, blinding of outcome assessment, incomplete outcome data, selective reporting, and other biases. Judgements (e.g., low, unclear, or high risk) are provided with supporting justifications for each domain. [file peerj-13-19537-s003.docx]

**Tabel S2.** Risk of bias assessment

1 (Swift et al., 2012)

| Bias | Authors’ judgement | Support for judgement |
| --- | --- | --- |
| Random sequence generation (selection bias) | Unclear risk | Randomized controlled trial compared a progressive combined—aerobic and resistance—exercise intervention with usual care |
| Allocation concealment (selection bias) | Unclear risk | A randomized 9-month exercise intervention with a control and a combination of aerobic and resistance training. Patients were randomly assigned to an intervention group after the baseline assessment |
| Blinding of participants and personnel (performance bias) All outcomes | High risk | The participants were not blinding |
| Blinding of outcome assessment (detection bias) All outcomes | Low risk | The assessor was blinding |
| Incomplete outcome data (attrition bias) All outcomes | Low risk | four patients in the control group lost to follow-up.  Two could not be contacted, and two were unwilling to participate.  In the intervention group, five patients were lost to follow-up.  One left for medical reasons, three could not be contacted, and one was unwilling to participate.  However, intention-to-treat analysis was applied |
| Selective reporting (reporting bias) | Low risk | Expected outcomes were reported |
| Other bias | Low risk | Other biases have not been identified |

2 (Ferrer-García et al., 2011)

| Bias | Authors’ judgement | Support for judgement |
| --- | --- | --- |
| Random sequence generation (selection bias) | Unclear risk | Eighty-four Spanish patients aged over 60 years were randomized to participate in a home-based, combined physical exercise program or standard treatment |
| Allocation concealment (selection bias) | Unclear risk | Patients enrolled in the intervention cohort followed the standard diabetes treatment and, a specific 24-week physical activity program. Consecutive probability sampling was performed, followed by the  randomization of each patient into one of the two cohorts. Eighty-four participants were selected and distributed into 2 groups |
| Blinding of participants and personnel (performance bias) All outcomes | Unclear risk | Information concerning the blinding of participants was not provided |
| Blinding of outcome assessment (detection bias) All outcomes | Unclear risk | Information concerning the blinding of the assessor was not provided |
| Incomplete outcome data (attrition bias) All outcomes | Unclear risk | Ten patients withdrew during the study, four from the intervention group and six from the control group.  The trials stated that most patients  who dropped out from the program did so for family reasons or due to difficulties in attending program learning sessions, however, the trial did not state all the reasons for patients dropped out.  Hence, intention-to-treat analysis was applied |
| Selective reporting (reporting bias) | Low risk | Expected outcomes were reported |
| Other bias | Low risk | Other biases have not been identified |

3 (Maiorana et al., 2002)

| Bias | Authors’ judgment | Support for judgment |
| --- | --- | --- |
| Random sequence generation (selection bias) | Unclear risk | Patients were randomized to an 8-week  exercise or non-training control group  . |
| Allocation concealment (selection bias) | Unclear risk | Patients were randomized to an 8-week  exercise or non-training control group |
| Blinding of participants and personnel (performance bias) All outcomes | Unclear risk | Information concerning the blinding of participants was not provided |
| Blinding of outcome assessment (detection bias) All outcomes | Low risk | The assessor was blinding |
| Incomplete outcome data (attrition bias) All outcomes | Unclear risk | One patient was unavailable for postexercise training assessment).  However, the reason for this was not explained. |
| Selective reporting (reporting bias) | Low risk | Expected outcomes were reported |
| Other bias | Low risk | Other biases have not been identified |

4 (Lambers et al., 2008)

| Bias | Authors’ judgment | Support for judgment |
| --- | --- | --- |
| Random sequence generation (selection bias) | Low risk | The randomization was performed using envelopes. |
| Allocation concealment (selection bias) | Low risk | Before commencing the first training, each  the patient had to choose a randomization envelope. The message inside stated that they were in a group  1 (combined exercise training), 2 (endurance training), or 3 (control group). |
| Blinding of participants and personnel (performance bias) All outcomes | Low risk | The participants were blinding |
| Blinding of outcome assessment (detection bias) All outcomes | Low risk | The assessor was blinding |
| Incomplete outcome data (attrition bias) All outcomes | Low risk | Seven patients dropped  out (two in the combination group, and five in the control group) due to non-compliance, illness, and loss of follow-up |
| Selective reporting (reporting bias) | Low risk | Expected outcomes were reported |
| Other bias | Low risk | Other biases have not been identified |

5 (Hale et al., 2022)

| Bias | Authors’ judgement | Support for judgement |
| --- | --- | --- |
| Random sequence generation (selection bias) | Low risk | A randomized, two-arm, parallel, open-label trial.  Randomization was based on lists generated using computer software (in Stata, using the malloc function) using  pseudo-random numbers and stratified by the center with  random block lengths (equally likely to be 2, 4, or 6) to  preserve allocation concealment. |
| Allocation concealment (selection bias) | Low risk | Each participant was randomly assigned to  either the exercise or control group (with an equal chance) by opening a sealed opaque envelope containing their allocation. The envelopes were prepared by an independent administrator based on lists prepared by the study  biostatistician. These lists were generated using computer software (in Stata, using the rallocfunction) using pseudo-random numbers and stratified by center with random block lengths (equally likely to be 2, 4, or 6) to preserve allocation concealment |
| Blinding of participants and personnel (performance bias) All outcomes | Low risk | The participants were blinding |
| Blinding of outcome assessment (detection bias) All outcomes | Low risk | The assessor was blinding |
| Incomplete outcome data (attrition bias) All outcomes | Low risk | Four patients dropped out (two in each group)  Because two of them were too busy and two feeling not well |
| Selective reporting (reporting bias) | Low risk | Expected outcomes were reported |
| Other bias | Low risk | Other biases have not been identified |

6 (Loimaala et al., 2003)

| Bias | Authors’ judgement | Support for judgement |
| --- | --- | --- |
| Random sequence generation (selection bias) | Unclear risk | 50 patients with type 2 diabetes were randomized into either a control group, in which they received conventional treatment only, or an exercise group |
| Allocation concealment (selection bias) | Low risk | Allocation concealment was based on a computer-generated randomization list stratified by gender in blocks of four and was used for the participants’ randomized assignment to ensure a close balance of the numbers in each group |
| Blinding of participants and personnel (performance bias) All outcomes | Unclear risk | Information concerning the blinding of participants was not provided |
| Blinding of outcome assessment (detection bias) All outcomes | Unclear risk | Information concerning the blinding of the assessor was not provided |
| Incomplete outcome data (attrition bias) All outcomes | Low risk | One subject from the exercise group  withdrew from the study because of lack of time. |
| Selective reporting (reporting bias) | Low risk | Expected outcomes were reported |
| Other bias | Low risk | Other biases have not been identified |

7 (Tessier et al., 2000)

| Bias | Authors’ judgement | Support for judgement |
| --- | --- | --- |
| Random sequence generation (selection bias) | Unclear risk | Forty-five patients were randomized into exercise or control group |
| Allocation concealment (selection bias) | Unclear risk | After baseline assessment, participants were randomized to exercise or control group |
| Blinding of participants and personnel (performance bias) All outcomes | Unclear risk | Information concerning the blinding of participants was not provided |
| Blinding of outcome assessment (detection bias) All outcomes | Unclear risk | Information concerning the blinding of the assessor was not provided |
| Incomplete outcome data (attrition bias) All outcomes | Low risk | five subjects in the experimental group and 1 subject  in the control group for personal reasons |
| Selective reporting (reporting bias) | Low risk | Expected outcomes were reported |
| Other bias | Low risk | Other biases have not been identified |

8 (Dunstan et al., 1998)

| Bias | Authors’ judgement | Support for judgement |
| --- | --- | --- |
| Random sequence generation (selection bias) | Low risk | Subjects were randomly assigned using block randomization to either an exercise (n=15) or a  non-exercise control group (n=12) for 8 weeks |
| Allocation concealment (selection bias) | Low risk | Participants were randomly assigned using block randomization to exercise or usual care after baseline testing using concealed randomization lists. |
| Blinding of participants and personnel (performance bias) All outcomes | Unclear risk | Information concerning the blinding of participants was not provided |
| Blinding of outcome assessment (detection bias) All outcomes | Unclear risk | Information concerning the blinding of the assessor was not provided |
| Incomplete outcome data (attrition bias) All outcomes | Low risk | Six subjects in the control group were unable to continue the study after the baseline period.  one required non-related surgery, another had a significant alteration in medication, and four had personal concerns and were unable to satisfactorily meet the time commitments of the study |
| Selective reporting (reporting bias) | Low risk | Expected outcomes were reported |
| Other bias | Low risk | Other biases have not been identified |

9 (Jorge et al., 2011)

| Bias | Authors’ judgement | Support for judgement |
| --- | --- | --- |
| Random sequence generation (selection bias) | Unclear risk | 48 individuals with type 2 diabetes mellitus were randomized to 4 groups: aerobic (n = 12), resistance (n = 12), combined (n = 12), and control (n = 12) |
| Allocation concealment (selection bias) | Unclear risk | Patients were randomly allocated into exercise or control groups after baseline assessment |
| Blinding of participants and personnel (performance bias) All outcomes | Unclear risk (Sabouri et al., 2021) | Information concerning the blinding of participants was not provided |
| Blinding of outcome assessment (detection bias) All outcomes | Unclear risk | Information concerning the blinding of the assessor was not provided |
| Incomplete outcome data (attrition bias) All outcomes | Unclear risk | Five patients dropped out of the study at the beginning of the trial: one patient dropped out because of health problems unrelated to the study, and the remaining 4 dropped out for private reasons. However, the trial did not state to which group they belonged. |
| Selective reporting (reporting bias) | Low risk | Expected outcomes were reported |
| Other bias | Low risk | Other biases have not been identified |

10 (Oliveira et al., 2012)

| Bias | Authors’ judgement | Support for judgement |
| --- | --- | --- |
| Random sequence generation (selection bias) | Unclear risk | Twenty-two patients were randomized into exercise or control group |
| Allocation concealment (selection bias) | Unclear risk | Twenty-two patients were randomly allocated into exercise or control groups after the baseline assessment |
| Blinding of participants and personnel (performance bias) All outcomes | Unclear risk | Information concerning the blinding of participants was not provided |
| Blinding of outcome assessment (detection bias) All outcomes | Unclear risk | Information concerning the blinding of the assessor was not provided |
| Incomplete outcome data (attrition bias) All outcomes | Unclear risk | One subject did not complete the study because of health problems unrelated to the investigation, and four subjects did not complete the study for personal reasons. However, the trial did not state to which group they belonged. |
| Selective reporting (reporting bias) | Low risk | Expected outcomes were reported |
| Other bias | Low risk | Other biases have not been identified |

11 (Sigal et al., 2007)

| Bias | Authors’ judgement | Support for judgement |
| --- | --- | --- |
| Random sequence generation (selection bias) | Low risk | A randomized, controlled trial. Participants were randomly allocated in equal numbers to exercise or control groups using block sizes varied randomly between 4 and 8. |
| Allocation concealment (selection bias) | Low risk | Central randomization was used, with allocation concealment before randomization, and block sizes varied randomly between 4 and 8. |
| Blinding of participants and personnel (performance bias) All outcomes | High risk | The participants were not blinding |
| Blinding of outcome assessment (detection bias) All outcomes | Low risk | The assessor was blinding |
| Incomplete outcome data (attrition bias) All outcomes | Low risk | Eight patients in the intervention group did not complete the study due to medical conditions and personal reasons.  Three patients in the control group did not complete the study because they were not satisfied with group allocation. |
| Selective reporting (reporting bias) | Low risk | Expected outcomes were reported |
| Other bias | Low risk | Other biases have not been identified |

12 (Gibbs et al., 2012)

| Bias | Authors’ judgement | Support for judgement |
| --- | --- | --- |
| Random sequence generation (selection bias) | Unclear risk | A total of 140 participants were randomized to either the exercise (70) or the control (70) |
| Allocation concealment (selection bias) | Unclear risk | A total of 140 participants were randomized to either the exercise (70) or the control (70) after baseline assessment. |
| Blinding of participants and personnel (performance bias) All outcomes | Unclear risk | Information concerning the blinding of participants was not provided |
| Blinding of outcome assessment (detection bias) All outcomes | Unclear risk | Information concerning the blinding of the assessor was not provided |
| Incomplete outcome data (attrition bias) All outcomes | Unclear risk | Twenty-one patients did not complete the study in the intervention group. Seven did not complete the study in the control group.  However, the trial did not state the reasons for the loss to follow-up. The only reason stated is that those with Losses to follow-up had a slightly worse cardiovascular profile compared to completers |
| Selective reporting (reporting bias) | Low risk | Expected outcomes were reported |
| Other bias | Low risk | Other biases have not been identified |

13 (Yavari et al., 2012)

| Bias | Authors’ judgement | Support for judgement |
| --- | --- | --- |
| Random sequence generation (selection bias) | Unclear risk | 80 patients were randomly assigned in equal numbers into exercise or control groups. |
| Allocation concealment (selection bias) | Unclear risk | 80 patients were randomly assigned in equal numbers into exercise or control groups after baseline assessment |
| Blinding of participants and personnel (performance bias) All outcomes | High risk | The participants were not blinding |
| Blinding of outcome assessment (detection bias) All outcomes | High risk | The assessors were not blinding |
| Incomplete outcome data (attrition bias) All outcomes | Unclear risk | The dropped-out cases were eliminated for the following reasons: 3 cases of added insulin therapy, 7 dropped out because of insufficient numbers of sessions, 2 dropped out due to repeated severe hypoglycemia in the first month of this protocol, 1 case of death due to a non-diabetic cause (car accident), and 2 cases of change of address. However, the trial did not state to which group they belonged |
| Selective reporting (reporting bias) | Low risk | Expected outcomes were reported |
| Other bias | Low risk | Other biases have not been identified |

14 (Cuff et al., 2003)

| Bias | Authors’ judgement | Support for judgement |
| --- | --- | --- |
| Random sequence generation (selection bias) | Unclear risk | A total of 28 obese postmenopausal women  with type 2 diabetes were randomly assigned to one of three 16-week treatments: control, aerobic  only training (Ae only), or aerobic plus resistance training (AE+RT) |
| Allocation concealment (selection bias) | Unclear risk | Patients were randomly allocated into exercise or control groups after baseline assessment |
| Blinding of participants and personnel (performance bias) All outcomes | Unclear risk | Information concerning the blinding of participants was not provided |
| Blinding of outcome assessment (detection bias) All outcomes | Unclear risk | Information concerning the blinding of the assessor was not provided |
| Incomplete outcome data (attrition bias) All outcomes | Low risk | All the participants completed the study. |
| Selective reporting (reporting bias) | Low risk | Expected outcomes were reported |
| Other bias | Low risk | Other biases have not been identified |

15 (Scheer et al., 2020)

| Bias | Authors’ judgement | Support for judgement |
| --- | --- | --- |
| Random sequence generation (selection bias) | High risk | 35 were allocated to the exercise or control group.  This study was not randomized in its final form, due to  the high drop-out rate from the control group in patients who were randomized in the initial blocks |
| Allocation concealment (selection bias) | Unclear risk | Patients were randomly allocated into exercise or control groups after baseline assessment |
| Blinding of participants and personnel (performance bias) All outcomes | Unclear risk | Information concerning the blinding of participants was not provided |
| Blinding of outcome assessment (detection bias) All outcomes | Low risk | the assessor was blinded |
| Incomplete outcome data (attrition bias) All outcomes | Low risk | Seven patients in the control group did not complete the study. Six withdrew and one undertook a formal exercise.  Two patients in the intervention group did not complete the study.  One discontinued intervention and one had a cancer diagnosis |
| Selective reporting (reporting bias) | Low risk | Expected outcomes were reported |
| Other bias | Low risk | Other biases have not been identified |

16 (Tan et al., 2012)

| Bias | Authors’ judgement | Support for judgement |
| --- | --- | --- |
| Random sequence generation (selection bias) | Unclear risk | 25 subjects (65.9 ± 4.2 yrs; M/F: 13/12) with a long history of type 2 diabetes (16.7 ± 6.7 yrs) were randomly allocated into either the exercise or control groups |
| Allocation concealment (selection bias) | Unclear risk | Patients were randomly allocated into exercise or control groups after baseline assessment |
| Blinding of participants and personnel (performance bias) All outcomes | Unclear risk | Information concerning the blinding of participants was not provided |
| Blinding of outcome assessment (detection bias) All outcomes | Unclear risk | Information concerning the blinding of the assessor was not provided |
| Incomplete outcome data (attrition bias) All outcomes | Low risk | Five subjects (three of the exercise group and two of the control group) dropped out of the study because of private reasons |
| Selective reporting (reporting bias) | Low risk | Expected outcomes were reported |
| Other bias | Low risk | Other biases have not been identified |

17 (Zarei et al., 2021)

| Bias | Authors’ judgement | Support for judgement |
| --- | --- | --- |
| Random sequence generation (selection bias) | Low risk | Twenty-four male patients with type 2 diabetes were randomized to two combined resistance aerobic exercise training group (n=12) and control group (n=12). Randomization was performed with a computer-generated randomized sequence of group allocation created. |
| Allocation concealment (selection bias) | Low risk | Patients were randomly allocated into exercise or control groups after baseline assessment, using randomization was performed with a computer-generated randomized sequence of group allocation created. |
| Blinding of participants and personnel (performance bias) All outcomes | Unclear risk | Information concerning the blinding of participants was not provided |
| Blinding of outcome assessment (detection bias) All outcomes | Unclear risk | Information concerning blinding of the assessor were not provided |
| Incomplete outcome data (attrition bias) All outcomes | Low risk | One patient in the intervention group did not complete the study.  Because did not complete the exercise session.  One patient in the control group did not complete the study.  Because traveling to another place |
| Selective reporting (reporting bias) | Low risk | Expected outcomes were reported |
| Other bias | Low risk | Other biases have not been identified |

18 (Magalhães et al., 2019)

| Bias | Authors’ judgement | Support for judgement |
| --- | --- | --- |
| Random sequence generation (selection bias) | Low risk | A randomized controlled trial included 96 participants with type 2 diabetes for a one-year supervised exercise intervention with three groups. Patients were randomly assigned to exercise or control group using the computer-generated list. |
| Allocation concealment (selection bias) | Low risk | Allocation of participants, a researcher external to the project used a computer-generated list of random numbers with an allocation ratio of 1:1:1 |
| Blinding of participants and personnel (performance bias) All outcomes | Low risk | Participants were blinding |
| Blinding of outcome assessment (detection bias) All outcomes | low risk | The assessors were blinding. |
| Incomplete outcome data (attrition bias) All outcomes | Low risk | Six patients in the intervention group dropped out.  Two changed the town, one had a myocardial infarction, one was unwilling to participate, and one was injured during training.  Five patients were in the control group.  Two were unwilling to participate, one changed the town and two changed medications |
| Selective reporting (reporting bias) | Low risk | Expected outcomes were reported |
| Other bias | Low risk | Other biases have not been identified |

19 (Magalhães et al., 2020)

| Bias | Authors’ judgement | Support for judgement |
| --- | --- | --- |
| Random sequence generation (selection bias) | Low risk | A randomized controlled trial included 96 participants with type 2 diabetes for a one-year supervised exercise intervention with three groups. Patients were randomly assigned to exercise or control group using a computer-generated list. |
| Allocation concealment (selection bias) | Low risk | Allocation of participants, a researcher external to the project used a computer-generated list of random numbers with an allocation ratio of 1:1:1 |
| Blinding of participants and personnel (performance bias) All outcomes | Low risk | Participants were blinding |
| Blinding of outcome assessment (detection bias) All outcomes | low risk | The assessors were blinding. |
| Incomplete outcome data (attrition bias) All outcomes | Low risk | Six patients in the intervention group dropped out.  Two changed the town, one had a myocardial infarction, one was unwilling to participate, and one was injured during training.  Five patients were in the control group.  Two were unwilling to participate, one changed their town and two changed medications |
| Selective reporting (reporting bias) | Low risk | Expected outcomes were reported |
| Other bias | Low risk | Other biases have not been identified |

20 (Annibalini et al., 2017)

| Bias | Authors’ judgement | Support for judgement |
| --- | --- | --- |
| Random sequence generation (selection bias) | Unclear risk | Sixteen male patients were randomly assigned to an intervention group (n = 8). |
| Allocation concealment (selection bias) | Unclear risk | Sixteen male patients were randomly assigned to an intervention group after the baseline assessment |
| Blinding of participants and personnel (performance bias) All outcomes | Unclear risk | Information concerning the blinding of participants was not provided |
| Blinding of outcome assessment (detection bias) All outcomes | Unclear risk | Information concerning the blinding of the assessor was not provided |
| Incomplete outcome data (attrition bias) All outcomes | Low risk | All the participants completed the study. |
| Selective reporting (reporting bias) | Low risk | Expected outcomes were reported |
| Other bias | Low risk | Other biases have not been identified |

21 (Sabouri et al., 2021).

| Bias | Authors’ judgement | Support for judgement |
| --- | --- | --- |
| Random sequence generation (selection bias) | Low risk | A total of 59 eligible subjects were categorized to  HIIT (n=16), ST [15], HIIT+ST (n=15), or CON (n=13), with an online randomization statistical service method using a computer-generated list. |
| Allocation concealment (selection bias) | Unclear risk | Patients were randomly assigned to an intervention group after the baseline assessment |
| Blinding of participants and personnel (performance bias) All outcomes | Unclear risk | Information concerning the blinding of participants was not provided |
| Blinding of outcome assessment (detection bias) All outcomes | Unclear risk | Information concerning the blinding of the assessor was not provided |
| Incomplete outcome data (attrition bias) All outcomes | Low risk | All the participants completed the study. |
| Selective reporting (reporting bias) | Low risk | Expected outcomes were reported |
| Other bias | Low risk | Other biases have not been identified |

22. (Motahari Rad et al., 2023)

| Bias | Authors’ judgement | Support for judgement |
| --- | --- | --- |
| Random sequence generation (selection bias) | Low risk | The subjects were randomly assigned to A-R (n ¼ 17), R-A (n ¼ 17), or non-exercise control (CON, n ¼ 17) groups, with an allocation ratio of 1:1:1. The randomisation was carried out through random number production after all baseline assessments had been completed. |
| Allocation concealment (selection bias) | Low | The subjects were randomly assigned to A-R (n ¼ 17), R-A (n ¼ 17), or non-exercise control (CON, n ¼ 17) groups, with an allocation ratio of 1:1:1. The randomisation was carried out through random number production after all baseline assessments had been completed |
| Blinding of participants and personnel (performance bias) All outcomes | Unclear risk | Information concerning the blinding of participants was not provided |
| Blinding of outcome assessment (detection bias) All outcomes | Low risk | The assessor was blinding |
| Incomplete outcome data (attrition bias) All outcomes | Low risk | All the participants completed the study. |
| Selective reporting (reporting bias) | Low risk | Expected outcomes were reported |
| Other bias | Low risk | Other biases have not been identified |

The subjects were randomly assigned to A-R (n ¼ 17), R-A (n ¼ 17), or non-exercise control (CON, n ¼ 17) groups, with an allocation ratio of 1:1:1. The randomisation was carried out through random number production after all baseline assessments had been completed.
