# Supplementary material for: Impact of concurrent aerobic and resistance training on body composition, lipid metabolism and physical function in patients with type 2 diabetes and overweight/obesity: a systematic review and meta-analysis
Source: PeerJ. 2025 Jun 11;13:e19537. doi: 10.7717/peerj.19537 (PMC12166852; doi:10.7717/peerj.19537)
Supplement: Supplemental Information 4 — A certainty assessment of the evidence for various outcomes (e.g., body mass, body fat, lipid profile, fasting blood glucose, and physical function). Certainty levels (very low, low, moderate) are determined based on factors such as risk of bias, inconsistency, indirectness, imprecision, and other considerations, with details on patient numbers and standardized mean differences (SMD) or mean differences (MD) for each outcome. [file peerj-13-19537-s004.docx]

**Table S3.** Summary of quality assessment findings (GRADE)

| Outcome | Certainty assessment | | | | | | | № of patients | | Effect | Certainty |
| --- | --- | --- | --- | --- | --- | --- | --- | --- | --- | --- | --- |
|  | N (studies) | Study design | Risk of bias | Inconsistency | Indirectness | Imprecision | Other considerations | CART | ST | Absolute (95% CI) |  |
| Body mass | 14 | RCT | Serious ^e, g^ | very serious ^d^ | not serious | Serious ^c^ | none | 400 | 361 | SMD **0.74 higher** (0.2 lower to 1.67 higher) | ⨁◯◯◯ Very low |
| Body mass  (≤16 weeks) | 11 | RCT | Serious ^e,g^ | very serious ^d^ | not serious | Serious ^c^ | none | 320 | 286 | SMD **1.33 higher** (0.06 higher to 2.6 higher) | ⨁◯◯◯ Very low |
| Body mass  (>16 weeks) | 3 | RCT | not serious | not serious | not serious | Serious ^c^ | none | 80 | 75 | SMD **0.23 lower** (0.54 lower to 0.09 higher) | ⨁⨁⨁◯ Moderate |
| Body fat | 9 | RCT | Serious ^e^ | not serious | not serious | Serious ^c^ | none | 290 | 261 | SMD **0.32 lower** (0.55 lower to 0.09 lower) | ⨁⨁◯◯ Low |
| Waist-to-hip ratio | 5 | RCT | Serious ^a^ | Serious ^b^ | not serious | Serious ^c^ | none | 65 | 66 | SMD **0.18 lower** (0.66 lower to 0.3 higher) | ⨁◯◯◯ Very low |
| Fat mass | 3 | RCT | not serious | not serious | not serious | Serious ^c^ | none | 85 | 83 | SMD **0.19 lower** (0.5 lower to 0.11 higher) | ⨁⨁⨁◯ Moderate |
| Lean body mass | 3 | RCT | not serious | not serious | not serious | Serious ^c^ | none | 77 | 102 | SMD **0.02 lower** (0.33 lower to 0.3 higher) | ⨁⨁⨁◯ Moderate |
| High-density lipoprotein | 11 | RCT | Serious ^e^ | Serious ^f^ | not serious | Serious ^c^ | none | 297 | 279 | SMD **0.44 higher** (0.05 higher to 0.82 higher) | ⨁◯◯◯ Very low |
| Low-density lipoprotein | 11 | RCT | Serious ^e,h^ | Serious ^f^ | not serious | Serious ^c^ | none | 300 | 289 | SMD **0.32 lower** (0.62 lower to 0.02 lower) | ⨁◯◯◯ Very low |
| Triglycerides | 12 | RCT | Serious ^e,h^ | not serious | not serious | Serious ^c^ | none | 321 | 305 | SMD **0.48 lower** (0.71 lower to 0.24 lower) | ⨁⨁◯◯ Low |
| Total cholesterol | 11 | RCT | Serious ^e,h^ | not serious | not serious | Serious ^c^ | none | 195 | 181 | SMD **0.35 lower** (0.58 lower to 0.12 lower) | ⨁⨁◯◯ Low |
| Fasting blood glucose | 9 | RCT | Serious ^g^ | Serious ^b^ | not serious | Serious ^c^ | none | 210 | 177 | SMD **0.4 lower** (0.74 lower to 0.07 lower) | ⨁⨁◯◯ Low |
| Physical function (aerobic capacity) | 2 | RCT | not serious | not serious | not serious | Serious ^c^ | none | 34 | 26 | MD **78.78 higher** (46.3 higher to 111.25 higher) | ⨁⨁⨁◯ Moderate |
| Physical function  (lower body strength) | 2 | RCT | not serious | Serious ^b^ | not serious | Serious ^c^ | none | 43 | 41 | MD **5.19 higher** (1.8 higher to 8.59 higher) | ⨁⨁◯◯ Low |

*CI, confidence intervals; MD, mean difference; SMD, standardized mean differences; RCT, randomized control trials.*

^a^ the study *was not randomized in its final form due to the high drop-out rate,*

^b^ there is moderate heterogeneity in the involved studies,

^c^ the included studies recorded a small sample size for both the control and intervention groups,

^d^ there is considerable heterogeneity in the studies,

^e^ information regarding the blinding of the participant and the assessor was not provided,

^f^ there is substantial heterogeneity in the study’s outcome,

^g^ blinding was not feasible for participants and
