# Supplementary material for: Impact of concurrent aerobic and resistance training on body composition, lipid metabolism and physical function in patients with type 2 diabetes and overweight/obesity: a systematic review and meta-analysis
Source: PeerJ. 2025 Jun 11;13:e19537. doi: 10.7717/peerj.19537 (PMC12166852; doi:10.7717/peerj.19537)
Supplement: Supplemental Information 5 — The studies excluded from the systematic review after full-text assessment, along with the specific reasons for exclusion, such as unrelated outcome measures, combined diet and exercise interventions, lack of a control group, inclusion of both diabetic and non-diabetic participants, or control groups performing exercise. [file peerj-13-19537-s005.docx]

**Table S4.** Excluded full-text articles with reasons.

| **Study** | **Reason** |
| --- | --- |
| Jennings et al. (2009) | No related outcome measures |
| [Legaard et al., 2022](#_ENREF_33)  [Bogardus et al., 1984](#_ENREF_7) | Combined diet and exercise intervention |
| (Adamo et al., 2005, Newton Jr et al., 2020, Sertbas et al., 2021, Larose et al., 2012, Tokmakidis et al., 2004) | No control group |
| Bello et al. 2014;  [Schreuder, Van Den Munckhof, Poelkens, Hopman, & Thijssen, 2015](#_ENREF_51) | Diabetics and non-diabetic patients were enrolled |
| [Boudou, De Kerviler, Erlich, Vexiau, & Gautier, 2001](#_ENREF_8)  [Kadoglou et al., 2013](#_ENREF_24)  [Wallace, Mills, & Browning, 1997](#_ENREF_62) | The control group performed an exercise |
