# Supplementary material for: Impact of concurrent aerobic and resistance training on body composition, lipid metabolism and physical function in patients with type 2 diabetes and overweight/obesity: a systematic review and meta-analysis
Source: PeerJ. 2025 Jun 11;13:e19537. doi: 10.7717/peerj.19537 (PMC12166852; doi:10.7717/peerj.19537)
Supplement: Supplemental Information 6 — The additional reports derived from original studies and specifies the reasons for their inclusion in the systematic review, based on the reported parameters such as body fat, body weight, fasting blood glucose, LDL-C, and HDL-C. [file peerj-13-19537-s006.docx]

**Table S5:** The subsequent reports of original studies

| Original Studies | Reports of original studies | Reason for inclusion (reported parameters) |
| --- | --- | --- |
| Jorge et al. (2011) | Oliveira et al. (2012) | Body fat, body weight, fasting blood glucose, LDL-C and HDL-C. |
| (Magalhães et al., 2019) | Magalhães et al. (2020) | Fasting blood glucose |
