# Supplementary figures and images for: Impact of concurrent aerobic and resistance training on body composition, lipid metabolism and physical function in patients with type 2 diabetes and overweight/obesity: a systematic review and meta-analysis
Source: PeerJ. 2025 Jun 11;13:e19537. doi: 10.7717/peerj.19537 (PMC12166852; doi:10.7717/peerj.19537)

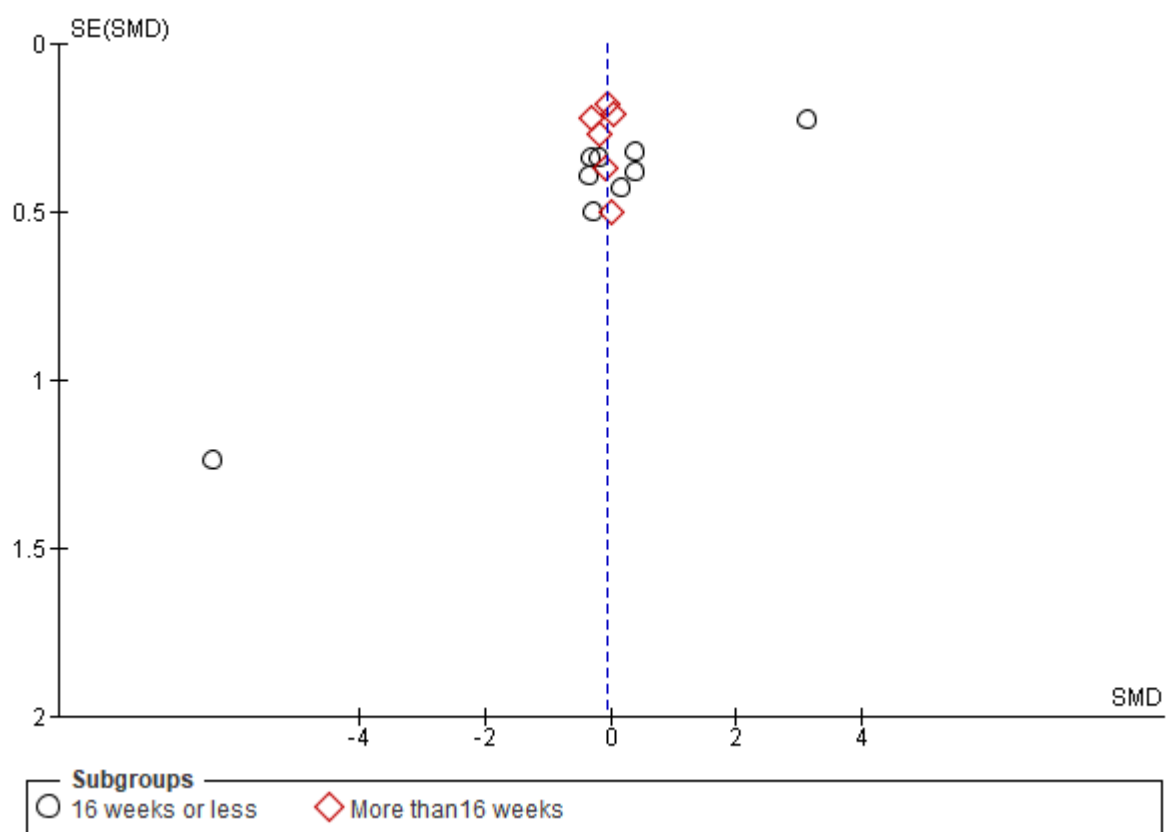

**Figure S1.** Forest plot of the effects of CART on BMI.

Supplement: Supplemental Information 7 [file peerj-13-19537-s007.pdf]

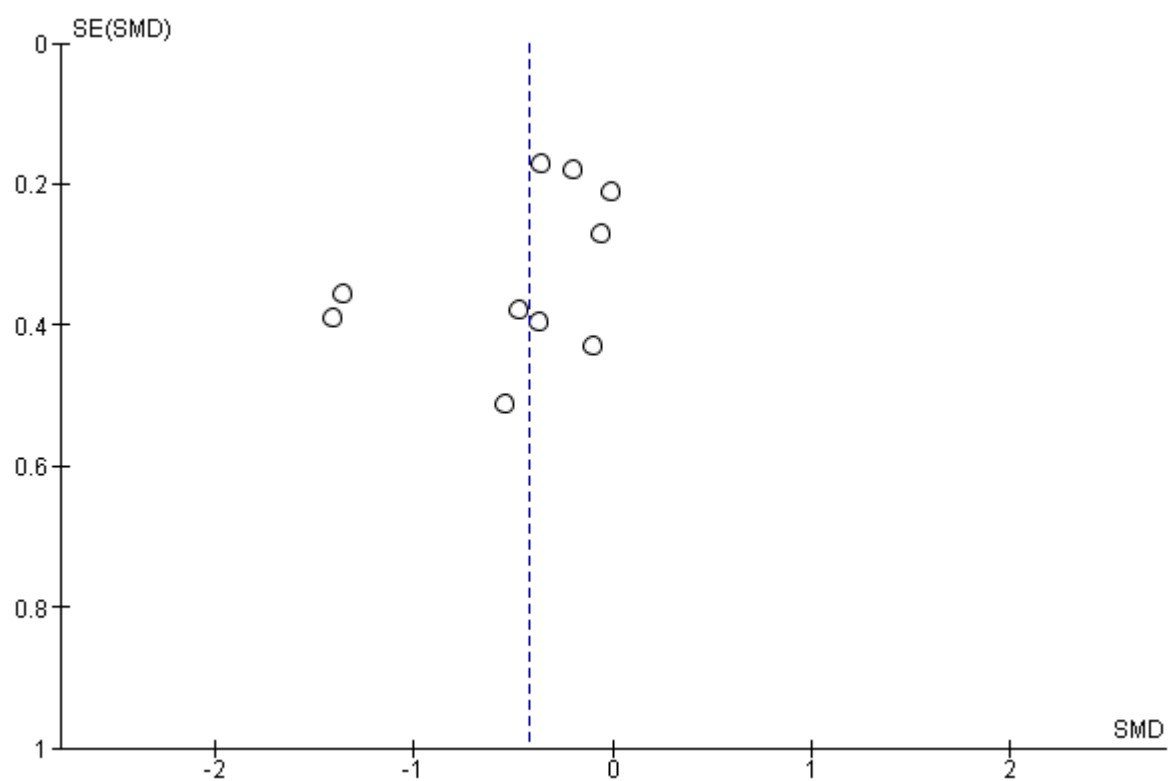

**Figure S2.** Forest plot of the effects of CART on body fat.

Supplement: Supplemental Information 8 — Forest plot of the effects of Concurrent Aerobic and Resistance Training (CART) on body fat, based on a meta-analysis of the included studies. [file peerj-13-19537-s008.pdf]

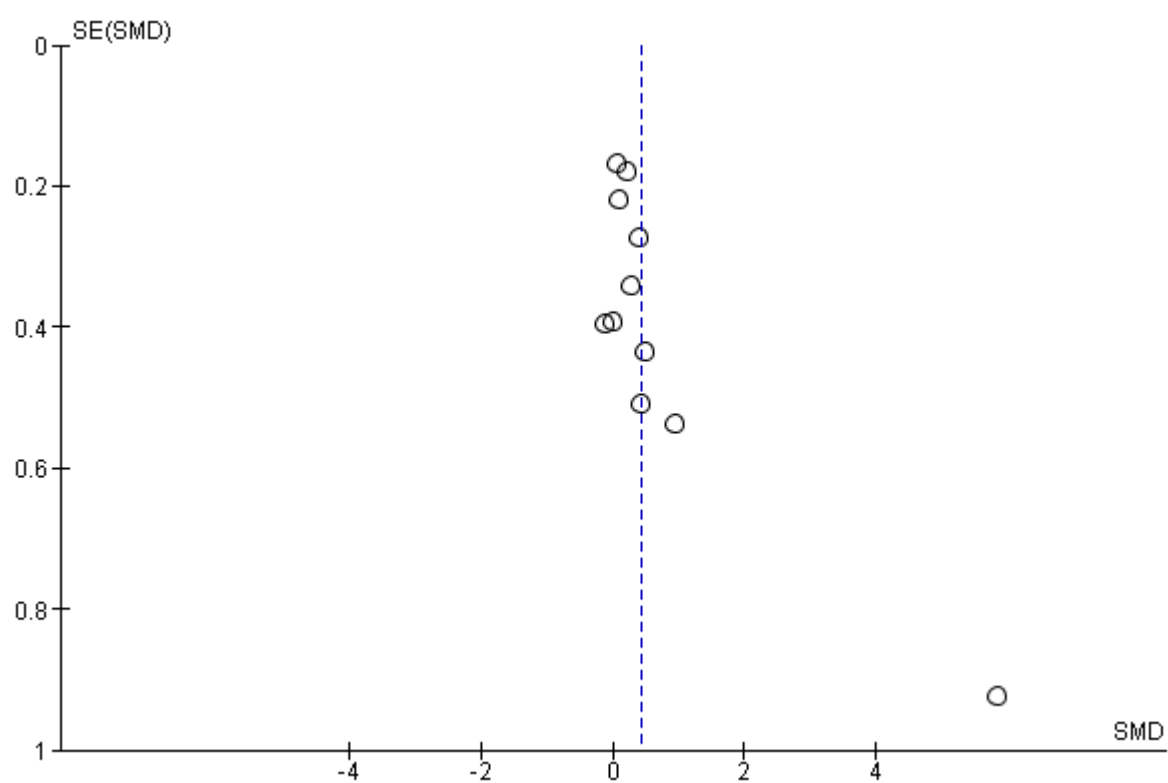

**Figure S3.** Forest plot of the effects of CART on HDL-C.

Supplement: Supplemental Information 9 — Forest plot of the effects of Concurrent Aerobic and Resistance Training (CART) on HDL-C levels, based on a meta-analysis of the included studies. [file peerj-13-19537-s009.pdf]

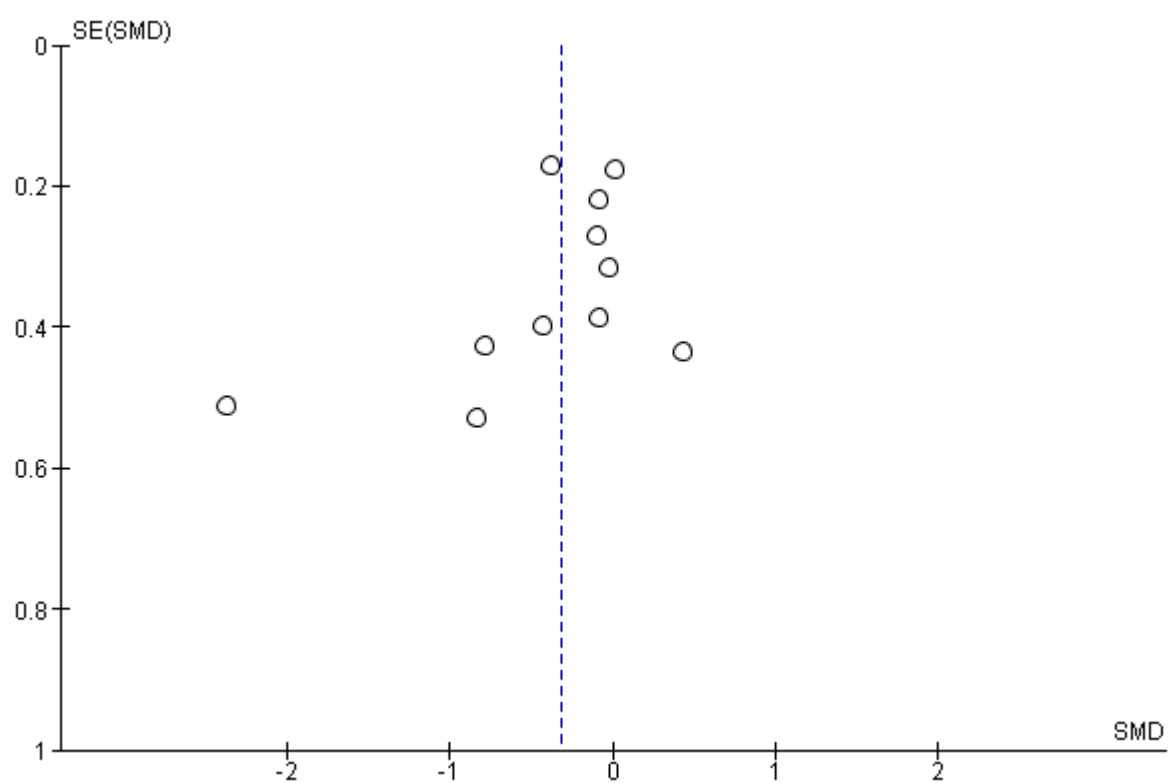

**Figure S4.** Forest plot of the effects of CART on LDL-C.

Supplement: Supplemental Information 10 — Forest plot of the effects of Concurrent Aerobic and Resistance Training (CART) on LDL-C levels, based on a meta-analysis of the included studies [file peerj-13-19537-s010.pdf]

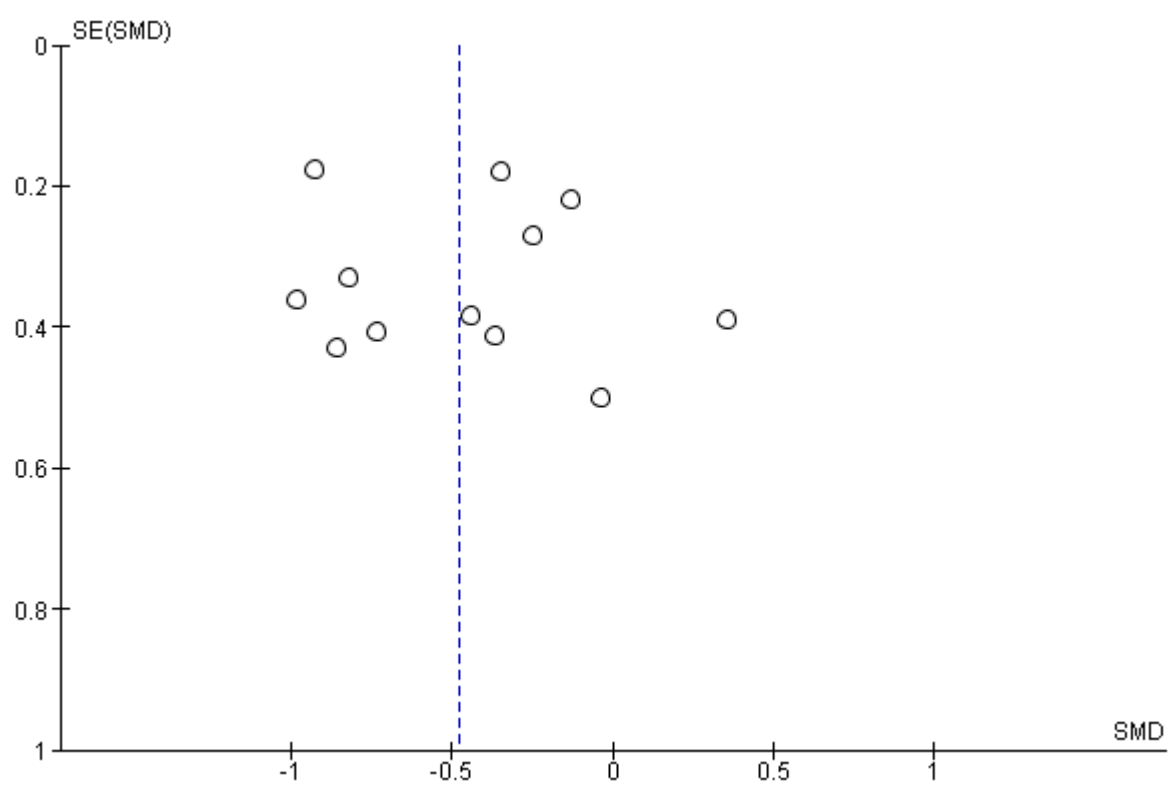

**Figure S5.** Forest plot of the effects of CART on TG.

Supplement: Supplemental Information 11 — Forest plot of the effects of Concurrent Aerobic and Resistance Training (CART) on triglyceride (TG) levels, based on a meta-analysis of the included studies. [file peerj-13-19537-s011.pdf]

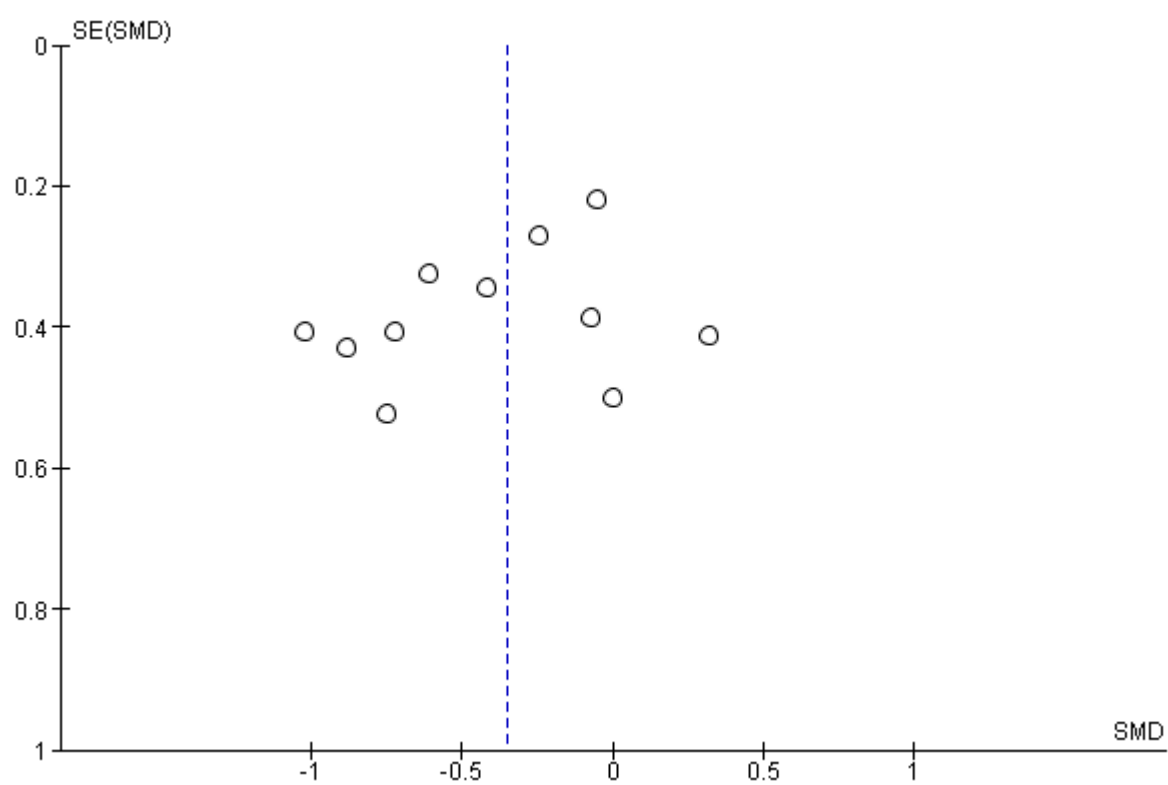

**Figure S6.** Forest plot of the effects of CART on TC.

Supplement: Supplemental Information 12 — Forest plot of the effects of Concurrent Aerobic and Resistance Training (CART) on total cholesterol (TC) levels, based on a meta-analysis of the included studies. [file peerj-13-19537-s012.pdf]
