# Supplementary material for: Impact of concurrent aerobic and resistance training on body composition, lipid metabolism and physical function in patients with type 2 diabetes and overweight/obesity: a systematic review and meta-analysis
Source: PeerJ. 2025 Jun 11;13:e19537. doi: 10.7717/peerj.19537 (PMC12166852; doi:10.7717/peerj.19537)
Supplement: Supplemental Information 13 — Based on 20 studies (N = 1,289), CART positively impacted body composition (↓ body fat, ↑ lean body mass), muscular fitness (↑ strength), glycolipid metabolism (↓ fasting blood glucose, ↓ total cholesterol, ↓ LDL-C, ↑ HDL-C, ↓ triglycerides), and cardiorespiratory fitness (↑ 6-min walk test). The average participant age was 57 ± 7 years, with a BMI of 31.1 ± 4.6 kg/m². [file peerj-13-19537-s013.pdf]

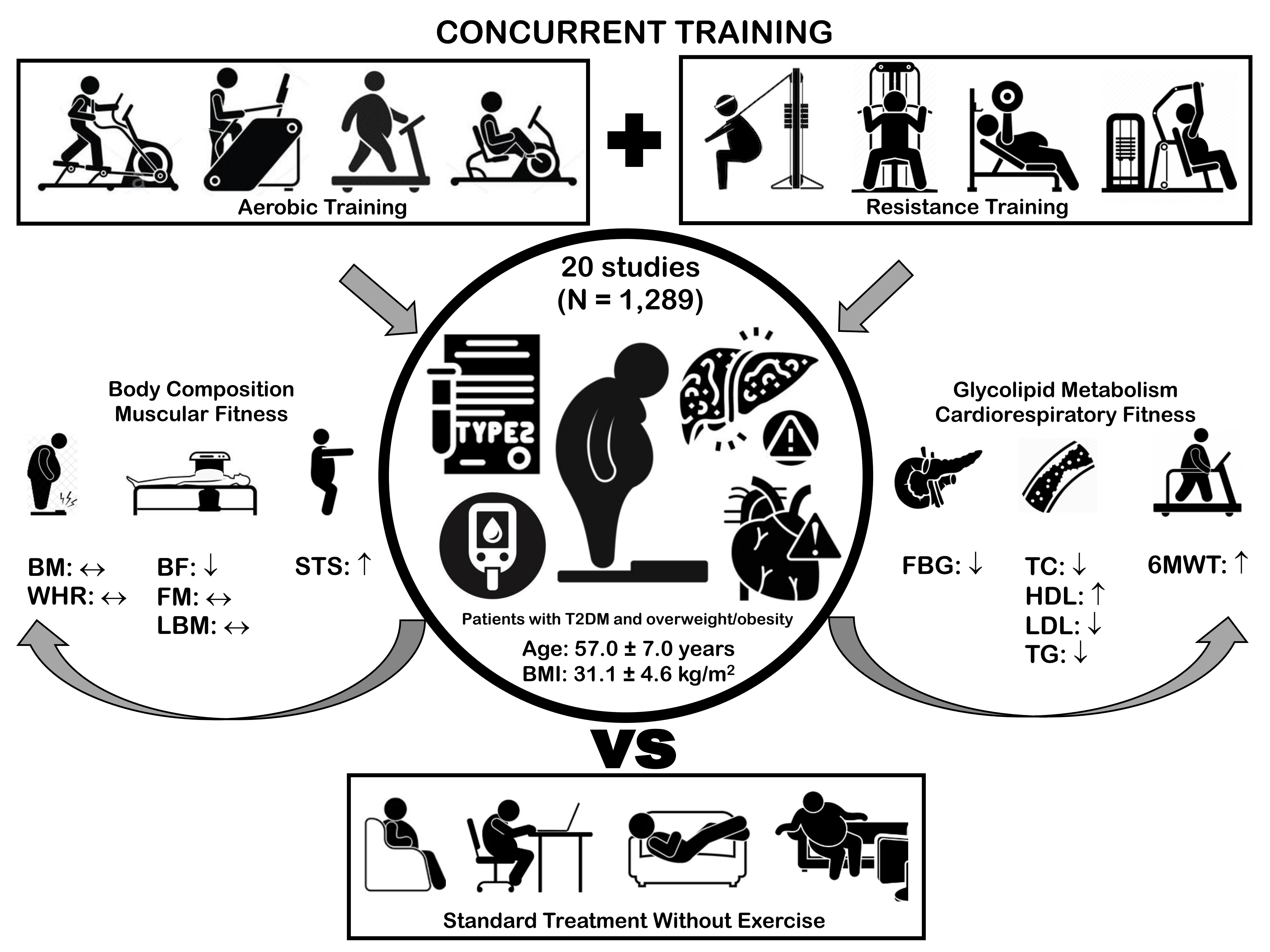
CONCURRENT TRAINING
Aerobic Training
Resistance Training
20 studies
(N = 1,289)
TYPE2
Body Composition
Muscular Fitness
Glycolipid Metabolism
Cardiorespiratory Fitness
BM: ↔
WHR: ↔
BF: ↓
FM: ↔
LBM: ↔
STS: ↑
FBG: ↓
TC: ↓
HDL: ↑
LDL: ↓
TG: ↓
6MWT: ↑
Patients with T2DM and overweight/obesity
Age: 57.0 ± 7.0 years
BMI: 31.1 ± 4.6 kg/m²
VS
Standard Treatment Without Exercise
